# Effects of Clinically Relevant MPL Mutations in the Transmembrane Domain Revealed at the Atomic Level through Computational Modeling

**DOI:** 10.1371/journal.pone.0023396

**Published:** 2011-08-17

**Authors:** Tai-Sung Lee, Hagop Kantarjian, Wanlong Ma, Chen-Hsiung Yeh, Francis Giles, Maher Albitar

**Affiliations:** 1 BioMaPS Institute, Department of Chemistry and Chemical Biology, Rutgers, The State University of New Jersey, Piscataway, New Jersey, United States of America; 2 University of Texas M.D. Anderson Cancer Center, Houston, Texas, United States of America; 3 Quest Diagnostics Nichols Institute, San Juan Capistrano, California, United States of America; 4 Cancer Therapy and Research Center and University of Texas Health Science Center, San Antonio, Texas, United States of America; National Institutes of Health, United States of America

## Abstract

**Background:**

Mutations in the thrombopoietin receptor (MPL) may activate relevant pathways and lead to chronic myeloproliferative neoplasms (MPNs). The mechanisms of MPL activation remain elusive because of a lack of experimental structures. Modern computational biology techniques were utilized to explore the mechanisms of MPL protein activation due to various mutations.

**Results:**

Transmembrane (TM) domain predictions, homology modeling, *ab initio* protein structure prediction, and molecular dynamics (MD) simulations were used to build structural dynamic models of wild-type and four clinically observed mutants of MPL. The simulation results suggest that S505 and W515 are important in keeping the TM domain in its correct position within the membrane. Mutations at either of these two positions cause movement of the TM domain, altering the conformation of the nearby intracellular domain in unexpected ways, and may cause the unwanted constitutive activation of MPL's kinase partner, JAK2.

**Conclusions:**

Our findings represent the first full-scale molecular dynamics simulations of the wild-type and clinically observed mutants of the MPL protein, a critical element of the MPL-JAK2-STAT signaling pathway. In contrast to usual explanations for the activation mechanism that are based on the relative translational movement between rigid domains of MPL, our results suggest that mutations within the TM region could result in conformational changes including tilt and rotation (azimuthal) angles along the membrane axis. Such changes may significantly alter the conformation of the adjacent and intrinsically flexible intracellular domain. Hence, caution should be exercised when interpreting experimental evidence based on rigid models of cytokine receptors or similar systems.

## Introduction

The myeloproliferative leukemia virus oncogene (MPL) encodes the thrombopoietin receptor, the major regulator of megakaryocytopoiesis and platelet formation [1], [2]. The MPL thrombopoietin receptor is a 635–amino acid protein consisting of two extracellular cytokine receptor domains, a transmembrane domain, and an intracellular domain containing two cytokine receptor box motifs [3]. Upon binding of thrombopoietin to the extracellular domain, MPL undergoes significant conformational changes and homodimerization. This conformational event induces phosphorylation of the intracellular non-receptor kinase partner of MPL, Janus kinase 2 (JAK2), initiating the downstream cascades critical in megakaryocyte and platelet formation [4].

Although JAK2 mutations at amino acid 617 and nearby positions account for the majority of patients with various forms of myeloproliferative neoplasms (MPNs), a substantial proportion of MPN patients lack a JAK2 mutation. Some JAK2 mutation-negative patients with essential thrombocytopenia and myelofibrosis have been reported to have MPL mutations, primarily at codons 515 and, to a lesser extent, 505 [5], [6], [7], [8], [9], [10]. A lack of experimental structures has hindered understanding of the activation mechanisms of MPL at the atomic level, and the detailed mechanisms of MPL mutational effects remain elusive.

We have demonstrated that modern computational approaches can be used to explore the structures and dynamics of biological molecules, even without experimental structures [11], [12]. In fact, computational approaches sometimes can provide information, especially insights into dynamical behaviors, which crystal structures cannot afford. Our previous work revealed possible origins of the effects of various JAK2 mutations through a series of molecular dynamics simulations starting with a homology model. Although not experimentally proven yet, the simulation-derived mechanisms are consistent with all available experimental and clinical evidence [12].

In this paper we applied computational biology approaches including, transmembrane domain prediction, homology modeling, and *ab inito* structure prediction, to obtain possible structural models of MPL. The structural models then served as starting points for large-scale long-time molecular dynamics simulations. Possible origins of the effects of MPL mutations at positions 505 and 515 were consolidated. This work represents the first published attempt to understand pathological effects of mutations of cytokine receptors at the atomic level.

## Methods

The following sections provide detailed descriptions of the computational approaches employed to identify the possible transmembrane region of MPL, build the homology model of the transmembrane region, predict the intracellular region structure, and merge individual domains to build the final structure; the setups for molecular dynamics simulations are also described. The wild-type and four clinically observed mutants were studied: W515L, W515K, S505N, and S505A.

### Transmembrane domain structure

Two transmembrane prediction servers were utilized to predict the MPL transmembrane domain: HMMTOP [13] predicted that residues 490 to 513 form the transmembrane domain while residues 486 to 490 and 514 to 518 are the interface regions; TMHMM [14] predicted that residues 490 to 512 form the transmembrane domain. Residues 490 to 512 were thus chosen as the transmembrane domain and were aligned with a known single-pass transmembrane protein segment (PDBId:2JPX). The extracellular part of MPL was removed except for residues 481 to 489, which were retained as a cap of the transmembrane domain. The alignment and the 2JPX structure were then used as the input template for the MODELLER program (9v7) [15] to build the homology model for the transmembrane region. Residues 513 to 517 were also included as the cap in the intracellular region.

### Intracellular domain structure

Despite the numerous homologues of MPL in the human genome, there is no known 3D structure of any homologue sequence for the MPL intracellular domain. To build the initial structure of the MPL intracellular domain from residues 517 to 635, we used a well-known *ab initio* protein prediction technique, Rosetta algorithm [16], running on the Robetta server [17].

### Merged structure

MODELLER was again used to build the merged structure. The homology model of the transmembrane domain and the intracellular domain predicted by the Rosetta *ab initio* method were used as the template structure for the merged structure. A distance constraint between L506 and F510 was set to ensure their experimentally observed hydrogen bond [18].

The merged structure then was inserted into the membrane, a bilayer of palmitoyloleoyl phosphatidylcholine (POPC) constructed by the VMD package, version 1.8.7 [19]. MPL was placed with a tilt angle of 0 degrees, and the alpha carbon of residue 501 was at the center of the membrane bilayer; any membrane molecules within 3 Å of MPL were removed.

### Molecular dynamics (MD) simulation setup

The MPL/membrane complex then was put in a 75 Å by 75 Å by 110 Å TIP3P water box [20] with the longest axis in the membrane bilayer normal direction. Sodium/chloride ions were added to reach the physiological concentration of sodium ions (0.14 M) and overall electric neutrality. The resulting system contained 59,249 atoms (MPL: 2,577; membrane: 19,028; water: 37,752; Na^+^: 17; Cl^−^: 17). All preparation steps were done using the VMD package.

All MD simulations were performed using the NAMD package (version 2.6) [21] with the CHARMM27 force field [22]. Default parameters and settings were used except as mentioned below. Periodic boundary conditions were used along with the isothermal-isobaric ensemble (NPT) at 1 atm and 310 K. The smooth particle mesh Ewald (PME) method [23] was employed with 75, 75, and 120 FFT grid points for the lattice directions x, y, and z, respectively. A cutoff of 12 Å for non-bonded interactions was used, with switching van der Waals potential beginning at 10 Å with the SHAKE algorithm [24] applied to bonds involving hydrogens.

The following procedure was used prior to the data collection (production) MD simulations. All heavy atoms of MPL were first restrained at their initial structure positions with a force constant of 50 kcal/mol/Å^2^. Membrane, water, and ion molecules were first energy-optimized and then underwent the following simulated annealing: the temperature was first increased from 0 K to 600 K at constant volume (1 K per ps) and held there for 500 ps, and then decreased from 600 K to 300 K at 1 K per ps and held at 300 K for 500 ps. The system then was kept at 300 K for 4 ns with constant pressure at 1 atm. The procedure was then repeated, resulting in an equilibration simulation of 10 ns for water, membrane molecules, and ions.

This membrane/water/ion-equilibrated system served as the starting point for the production simulations. Mutant systems were created with the VMD package on the basis of the equilibrated wild-type system; hence, all mutants have the same initial structure. One or two nearby cations/anions were removed to reach electric neutrality for mutants involving charge changes, with an additional 1 ns equilibration of membrane/water/ion. The restraint force constant then was gradually reduced to 3 kcal/mole/Å^2^ during a 500 ps period, followed by a 2,000-step energy minimization for all atoms and a 300-ps heating period with the temperature increased from 0 to 300 K at a rate of 1 K per ps.

In the production runs, a 140-ns MD simulation was performed for each mutant without any restraining potential applied. The last 100 ns of data were used for data analysis.

## Results and Discussion

**Figure 1:**
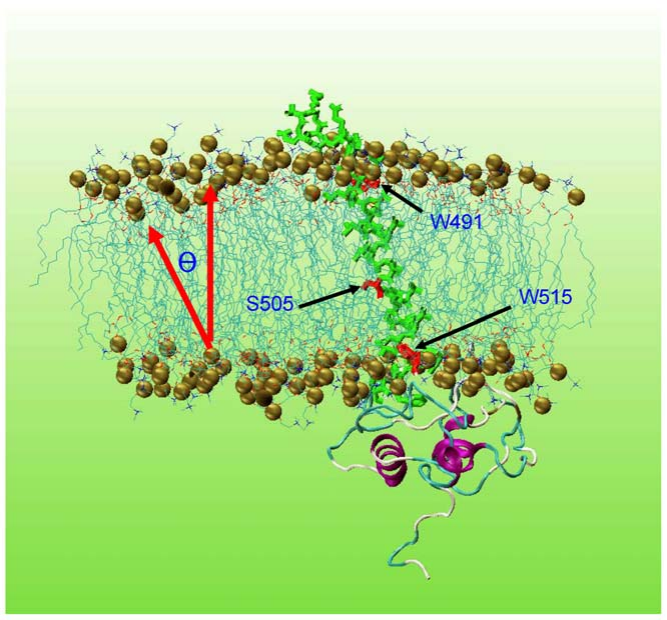
Snapshot of the simulation trajectory of wild-type MPL at 140 ns. The gold balls represent the phosphorus atoms of the membrane bilayer. Three key residues, W491, S505, and W515, are colored red and indicated by the black arrows. The upper part of the figures is the extracellular region while the lower part the intracellular region. The tilt angle θ, demonstrated by the red arrows, defines the tilt of the transmembrane domain relative to the membrane normal.

Since all mutations simulated in this work are located in the transmembrane region, we first analyzed the impact of these mutations on the position of the transmembrane domain. The snapshot of the simulation trajectory of the wild-type MPL at 140 ns is shown in Figure 1, which gives an overview of the simulation-derived MPL structures. Note that wild-type and mutant simulations have exactly the same starting structure, except for the protein side-chains of mutated positions.

### Hydrophobic/hydrophilic analysis

**Figure 2:**
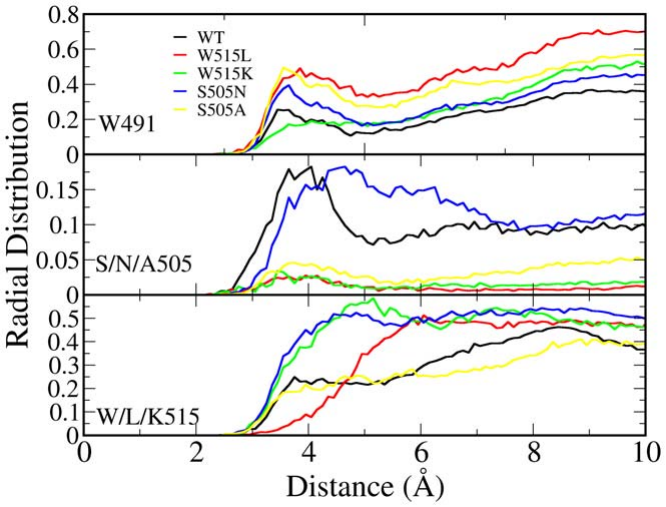
The radial distribution function (RDF, g(r) ) between water molecules and the C_β_ atoms of residues 491 (upper panel), 505 (middle panel), and 515 (lower panel) of MPL. The RDF is the distance distribution of two groups of atoms, calculated from the last 100 ns trajectory for each simulation with a sampling frequency of 10 ps, (i.e., 10,000 data points for each mutant).

Figure 2 shows the radial distribution function (RDF, g(r)) between water molecules and the C_β_ atoms of residues 491 (upper panel), 505 (middle panel), and 515 (lower panel). The RDF is the distribution of the distance between two groups of atoms. The RDF between water molecules and a protein residue can be treated as a measure of how deep the residue is buried from the solvent. Here it is also the measure of how deep a certain residue buried in the membrane. For residue 491, located near the extracellular region (Figure 1), the water RDF has similar shapes for all mutations. However, the magnitudes of the water RDF differ among mutants, which implies that the positions of the whole transmembrane domain were shifted. Residue 505 would be expected to be deeply buried in the membrane, since it is far from both the intracellular region and the intracellular region, based on the sequence. Nevertheless, the wild-type and S505N simulations both show significant water distribution around residue 505; the data imply that S505 and N505 both interact with water molecules from the intracellular region, with N505 having a stronger interaction with water. In fact both simulations revealed that the membrane had a small opening from time to time, allowing a few water molecules to enter the membrane and contact the residue 505 side-chain. Note that it is not possible to observe this type of interaction in a crystal structure. The polar side-chains of S505 and N505 are clearly the source of the interaction; S505A, which has a less polar side-chain, exhibits a much smaller water RDF. The polarity of residue 505 has been experimentally reported to be critical in the conformational changes of MPL [25]. W515L and W515K alter the position of the whole transmembrane region such that residue 505 is can no longer access the intracellular water molecules.

The water RDF for the residue 515 (Figure 2, lower panel) shows that W515 is located at the interface between the intracellular region and the membrane. This finding is consistent with other studies [26], [27] and suggests that tryptophan can serve as an interface anchor for membrane proteins. In fact, W491 and W515 could be the anchor points for the transmembrane domain for the extracellular and intracellular regions, respectively, as they have similar water RDF results. Nevertheless, W515K and S505N show larger water distributions than the wild-type MPL, suggesting that the transmembrane domain moves toward the intracellular region in both mutants. W515L has an opposite effect in that the RDF is smaller and the L515 residue is buried more deeply in the membrane. S505A has an RDF similar to that of the wild-type MPL, suggesting that this mutation does not have a significant effect on the position of W515.

### MPL transmembrane domain tilt angle relative to cell membrane

The above analysis of water RDF describes the water distribution around certain resides. Subsequently, we further identified the relative position changes of the MPL transmembrane domain by examining relevant geometrical variables.

The transmembrane tilt angle, defined in Figure 1, describes the degree of tilting of the transmembrane domain in cell membrane. The starting structures of wild-type and all MPL mutants have tilt angles of 0 degrees. Tilt angles derived from all simulations are listed in Table 1. The results suggest that, for MPL, the transmembrane tilt angle is significantly deviated from the starting structures (0 degrees), consistent with other studies [28], [29], [30], [31]. The RDF distributions in fact suggest that W491 and W515 are the anchor points on the membrane/water interfaces and may cause the observed ∼30-degree tilt angle for all simulations. Figure 1 also shows the positions of W491 and W515 relative to the membrane.

**Table 1:** MPL Transmembrane Domain Tilt Angles Derived from Molecular Dynamics Simulations.

Mutant	Tilt Angle ⊖, average (standard deviation)°
WT	27.6 (7.0)
W515L	28.8 (6.4)
W515K	25.1 (7.5)
S505A	21.9 (10.2)
S505N	33.5 (5.8)

⊖, angle of tilt of MPL transmembrane domain in relation to the membrane normal (Figure 1). Average values are calculated from the last 100 ns trajectory for each simulation, with a sampling frequency of 10 ps (i.e., 10,000 data points per mutant.

Although the tilt angles in simulations demonstrated large deviations (implying mobility of the transmembrane domain inside the membrane) and it is difficult to draw firm conclusions based solely on tilt angle statistics, S505A and S505N do appear to exhibit significant differences in tilt angles (21.9 and 33.5 degrees, respectively). This finding can be explained by the fact that A505 tends to be buried in the membrane and causes the transmembrane domain to be more aligned with the membrane normal direction, while N505 will have an opposite effect. Similar observations have been reported in other systems [30].

### TM rotation (azimuthal) angle

**Figure 3:**
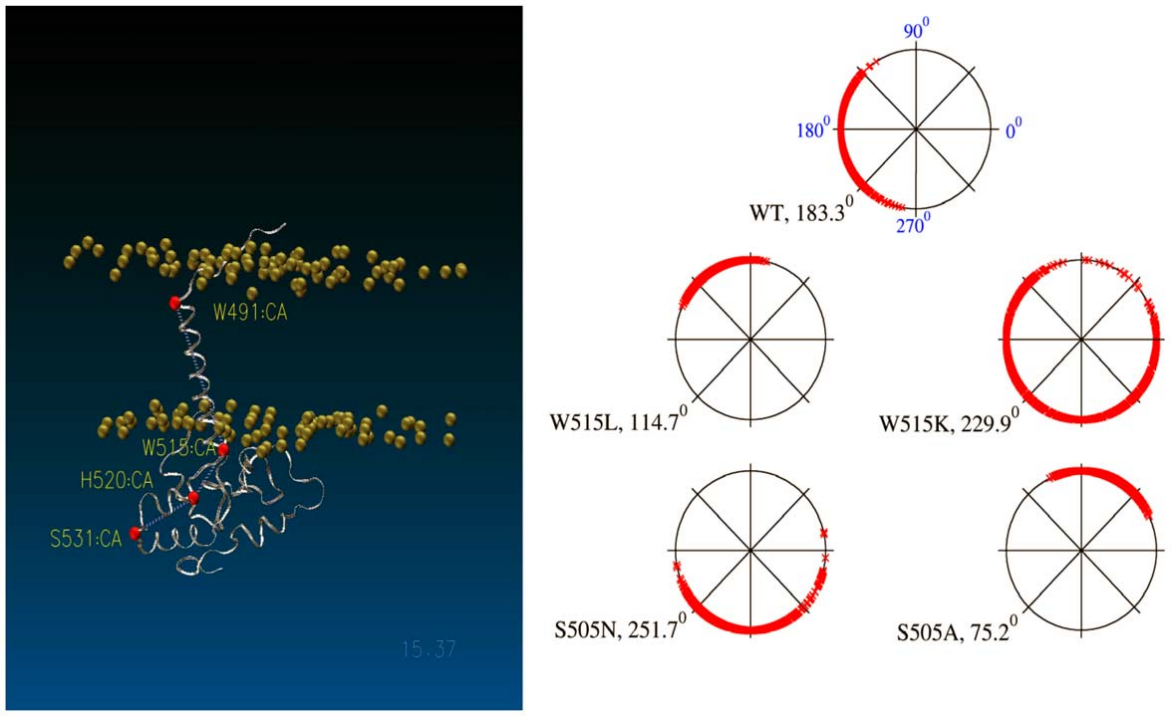
Effect of MPL mutations on rotation angle along the membrane axis (ie, the azimuthal angle). Left panel: depiction of the azimuthal angle, defined as the torsion between 4 C_α_ atoms (red dots) of the MPL intracellular domain. The gold balls represent the phosphorus atoms of the membrane bilayer. Right panel: distribution of azimuthal angle in all simulations; the average values are indicated next to the mutant name. The distribution is calculated from the last 100 ns trajectory for each simulation with a sampling frequency of 100 ps (i.e., 1,000 data points for each mutant).

The tilt angle alone is not enough to describe the relative position of the transmembrane region, which could also rotate relative to the intracellular domain. The averages of this rotation (azimuthal angle) and its distribution derived from simulations are shown in Figure 3. The starting structures of wild-type and all MPL mutants have azimuthal angles of 0 degrees. The average values and distributions of the azimuthal surprisingly vary across MPL mutants. This may imply that the junction between the transmembrane and intracellular domains is flexible and can be locked into different positions when the transmembrane domain moves along the membrane. This is especially true in the case of W515K because the charged side chain makes it very hydrophilic, causing the transmembrane domain to sometimes move toward the intracellular region. This movement causes the intracellular domain to undergo almost free rotation as the azimuthal angle distributes over the entire 360 degrees (Figure 3).

### The intracellular domain conformation is highly dependent on the transmembrane domain position

**Figure 4:**
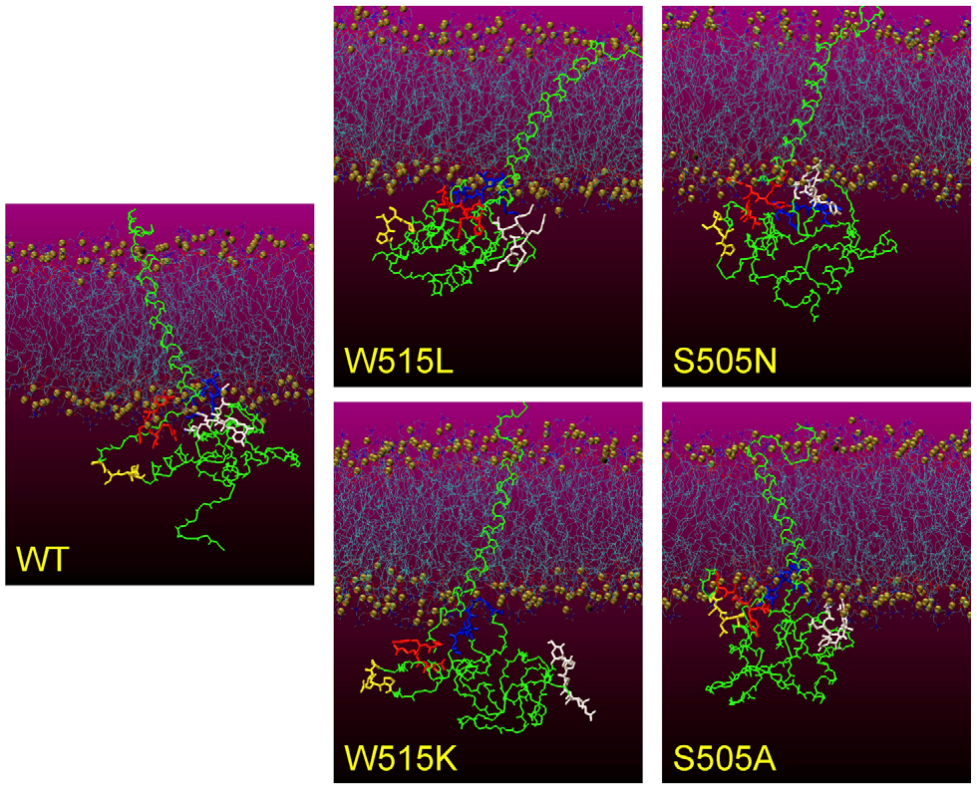
Snapshots showing the conformational changes of the intracellular (IC) domain due to different mutations. The gold balls represent the phosphorus atoms of the membrane bilayer. The MPL protein is colored green except for residues 50 to 53 (the Box1 motif, in yellow), 40 to 44 (red), 73 to 79 (blue), and 109 to 114 (white). Snapshots are taken from the simulation trajectories at 140 ns.

Whereas there is a consensus that MPL has a is a one-pass transmembrane membrane domain whose structure is most likely a single alpha helix, as we have seen in the simulations, there is no structural evidence for the intracellular domain. Although nuclear magnetic resonance (NMR) studies have provided experimental evidence on the hydrogen bonding patterns near the intracellular domain of MPL [18], the overall folding of the intracellular domain remains elusive. In this study the Rosetta *ab initio* protein structure prediction was used to build the initial structure of the intracellular domain. Although *ab initio* protein structure prediction techniques are maturing, we cannot yet reliably assess the quality of our model structure of the intracellular domain. Hence, the intracellular domain structures derived from our simulations (Figure 4) may not be used as reliable structural models. However, these simulations may be used to derive the impact of transmembrane domain movement on the intracellular domain structure.

Snapshots representing the conformational changes of the intracellular domain due to different mutations are shown in Figure 4. Three regions contacting the membrane (residues 40–44, 73–79, and 109–114) and the Box1 region (residues 50–53) responsible for JAK2 binding are highlighted with different colors. Relative to the wild-type simulation, all mutant simulations showed significant changes in the positions of these three contact regions in relation to the membrane. This is intriguing because the transmembrane and intracellular regions are usually thought of as individual domains; thus, the strong influence of transmembrane domain changes on the intracellular region was unexpected. This finding leads to an interesting issue: can separately crystallized intracellular domain structures represent the real structures when the interfaces between the transmembrane and intracellular domains have a large impact on the latter?

### Possible constitutive activation of JAK2 through MPL S505 and W515 mutations

With the limitations of currently available computational approaches, the intracellular domain structure reported here is putative and requires further experimental verification. Nevertheless, we examined the possible effects of MPL mutations on the Box1 loop of the intracellular domain. The Box1 loop is believed to be the JAK2 binding site [32]. Interestingly, Figure 4 shows that the Box1 loop (in yellow) is open only in the wild-type MPL. In all mutants, the Box1 loop is buried or packed with nearby residues. Although JAK2 was not included in our simulations, the conformational change of the Box1 loop will likely change the binding mode between MPL and JAK2. This could explain the constitutive activation of JAK2 due to S505 and W515 mutations. Hence, based on the disruption of the azimuthal angle and the impact on the structure of the intracellular domain, the predicted order of mutational effects is as follows: W515K > S505A >W515L > S505N; this predicted order needs to be experimentally verified.

### Possible mechanisms of activation of MPL/JAK2 signaling pathway

Traditionally the activation of membrane receptors and their partner kinases is explained by assuming that the individual domains are more-or-less rigid. Upon binding to the ligand, the receptor forms a dimer and the relative orientation between domains of a receptor dimer causes the activation processes [10], [18], [33], [34], [35], [36]. The intrinsic tilt angles of transmembrane domains have seldom been considered, and the azimuthal angles are usually totally ignored. It has been shown that the erythropoietin receptor has a juxtamembrane domain containing an essential and precisely oriented hydrophobic motif, and that insertion of extra residues may cause different azimuthal angles between the transmembrane and intracellular domains [37]. This may suggest that the azimuthal angles between the transmembrane and intracellular domains play critical roles in the signaling activation in similar systems. Our study shows that the MPL mutants cause significant changes in azimuthal angles and hence the intracellular domain conformations. Thus, it is possible that the control of azimuthal angles could be one of the major factors in signaling activation mechanisms for membrane receptors, as the azimuthal angle can be changed by rotating the transmembrane domain upon ligand binding as proposed in different systems [38]. The azimuthal angle apparently is determined by the conformation of the juxtamembrane domain, which has been recently demonstrated as the center stage of signal activations [18], [39], [40], [41].

Taken together, the above studies indicate that signal activation may involve the following events: ligand binding to the receptor causes rotation of the transmembrane domain and conformational changes of the juxtamembrane domain, which results in significant conformational changes of the intracellular domain. The anchor points of the transmembrane domains are thus critical to holding the transmembrane domain in the right tilt and azimuthal angles. Understanding of the detailed conformational changes may require dynamic descriptions rather than traditional rigid-domain models.

**Figure 5:**
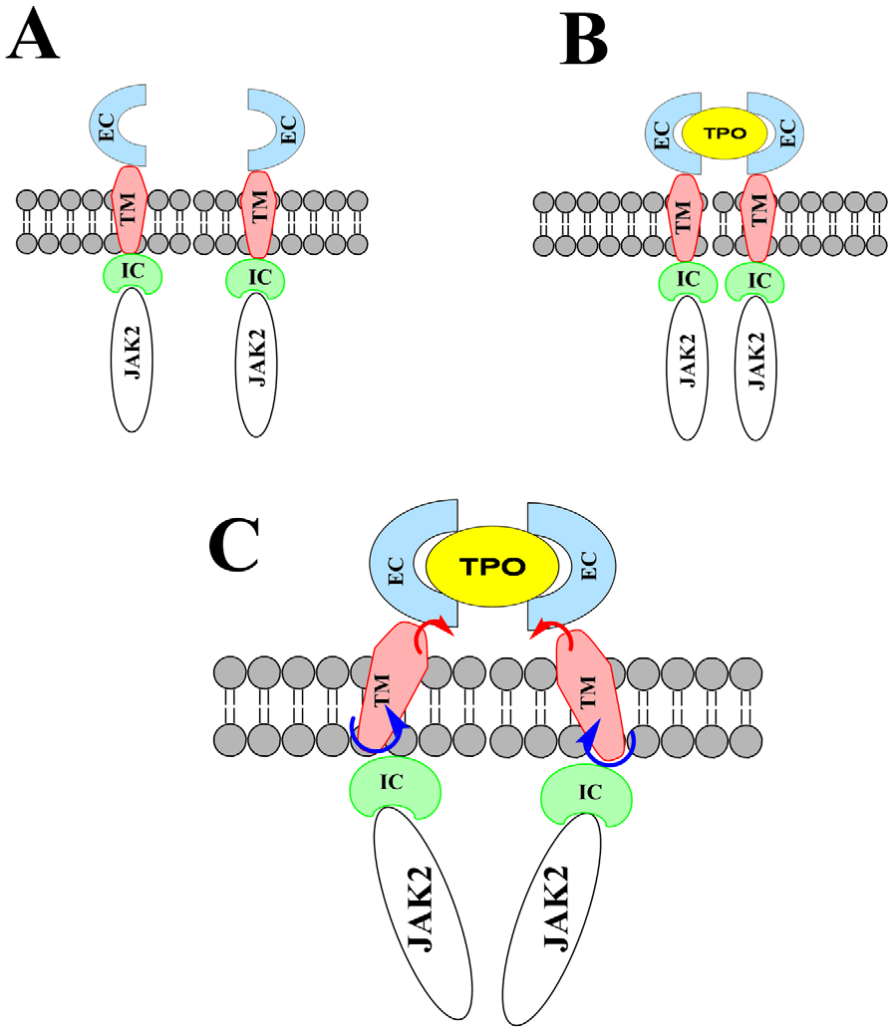
Schematic representations of the traditional view of activation of the MPL/JAK2 signaling pathway (from A to B), and a possible alternative view derived from the current work (from A to C). The A-to-B path shows that a ligand (thrombopoietin, TPO) binds to the extracellular (EC) domain MPL molecule, forming MPL dimers and bringing the transmembrane (TM) and intracellular (IC) domains together. The A-to-C path suggests that ligand binding changes the tilt (red arrows) and azimuthal (blue arrows) angles of the TM domain, resulting in conformational changes in the IC domains and the IC-JAK2 binding mode.

In contrast to most explanations for the activation mechanism, where the transmembrane and intracellular domains are usually treated as being rigid and the activation of JAK2 is explained by the relative translational movement between those rigid domains [10], [18], [33], [34], [35] (Figure 5, A-to-B), our results suggest that mutations within the transmembrane region could result in other types of conformational changes, including tilt angle and rotation (azimuthal) angle along the membrane axis (Figure 5, A-to-C). Such changes may significantly alter the conformation of adjacent IC domain. Hence we suggest that it may be necessary to consider these two angles when establishing molecular models for cytokine signaling events.

### Description of the dynamic behavior of membrane proteins is necessary

An important aspect of this study is that it highlights possible limitations of crystal structures for studying membrane proteins. All analyses reported here are based on long-time (140 ns for each mutant) molecular dynamics simulations that sample over different local and global conformations of different domains of MPL. The results cannot be obtained by static models. Although crystallography techniques are critically important in structural biology, crystal structures are averaged structures and cannot provide dynamic insights. For example, the rotation angle distribution analysis in the last section gives deep insights into how the intracellular domain may be influenced by the location of the transmembrane domain. This type of analysis is not possible with static crystal structures alone. To deeply understand the activation process in signaling transduction, other approaches such as NMR techniques and molecular dynamics simulations are necessary.

### Conclusion

We present the first full-scale molecular dynamic simulations on the wild-type and clinically observed mutants of the MPL transmembrane protein, a critical element of the related JAK2 signal pathway. Simulation results suggest that S505 and W515 are important in keeping the transmembrane domain in its correct position. Mutations at these two positions cause movement of the transmembrane domain, alter the conformation of the nearby intracellular domain in an unexpected way, and may cause constitutive activation of MPL's kinase partner, JAK2. We predict the order of impact of the mutational effects to be in the following sequence: W515K > S505A > W515L > S505N.

Our results suggest that mutations within the transmembrane region could cause conformational changes that are seldom considered, such as the tilt angle change and azimuthal rotational angle (the angle along the membrane normal) change. Such changes may significantly alter the conformation of the adjacent intracellular domain. Hence, extreme caution should be exercised when interpreting experimental evidence based on rigid models of cytokine receptors or similar systems.
